# Supplementary figures and images for: Comparative Serum Proteome Profiling of Canine Benign Prostatic Hyperplasia before and after Castration
Source: Animals (Basel). 2023 Dec 14;13(24):3853. doi: 10.3390/ani13243853 (PMC10740436; doi:10.3390/ani13243853)

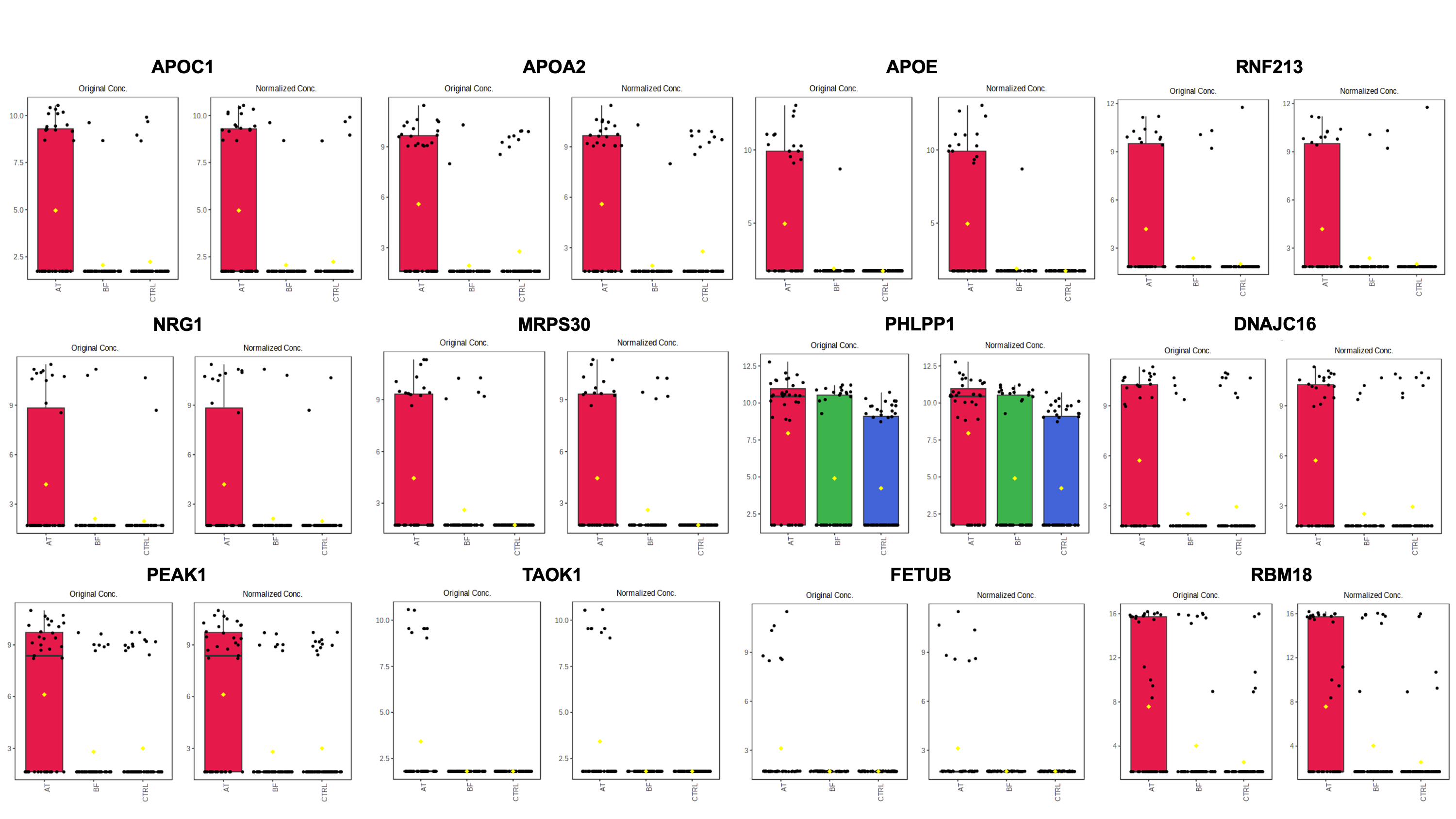

Supplement: Supplementary file 1 [file animals-13-03853-s001.zip › S. Figure 1 Boxplot of protein expression significantly different between AT (red), BF (green), and CTRL (blue) of APOC1, APOA2, APOE, RNF213, NRG1, MRPS30, PHLPP1, DNAJC16, PEAK1, TAOK1, FETUB, RBM18.tiff]
